# Complete Duplex of the Left Ureter with Lower Moiety Hydronephrosis Secondary to Ureteral Stone in Adult

**DOI:** 10.1155/2022/6552889

**Published:** 2022-04-12

**Authors:** Masresha S. Dino, Alemayehu Tegegn Tefera, Kaleab Hebtemichael Gebreselassie, Sena Sefera Akkasa, Ferid Ousman Mummed

**Affiliations:** St. Paul's Hospital Millennium Medical College, Department of Surgery, Urology Unit, Addis Ababa, Ethiopia

## Abstract

Ureteral duplication is a common embryologic abnormality of the kidney with an incidence rate of 0.8% in adults. However, complete duplex ureters opening independently into the urinary bladder are rarely present. We report a 35-year-old female who presented with left flank pain in the last three years. Abdominal CT scan showed left complete duplicated system with lower moiety hydroureteronephrosis and obstructed midureteral stone. The patient underwent left retroperitoneal exploration with complete excision of the hydronephrotic sac. The postoperative course was uneventful without complications. In conclusion, a complete duplex kidney with lower moiety hydronephrosis caused by mid ureteric stone is rare. The renal duplication system should be diagnosed and followed with image guidance periodically as the late diagnosis may have poor outcomes with loss of the kidney or part of it.

## 1. Introduction

Ureteral duplications are a common congenital anomaly of the kidney and the urinary tract system [1, 2]. It has an incidence rate of 0.8% in the healthy adult population, 0.3%–2.5% incidence in an autopsy, and 2–4% in patients investigated for urinary tract symptoms [3]. Complete duplication occurs in 40% of cases, whereas partial duplication occurs in 60% of the duplication [3].

Generally, duplex renal systems are asymptomatic and diagnosed incidentally; however, several conditions requiring treatment have been observed in association with duplex systems: obstruction, reflux disease, and urinary stones are not peculiar but may be commoner in duplex systems [4].

Concerning obstruction, the upper moiety may be at a greater risk caused by ureterocele and lower moiety is at risk of reflux nephropathy [5, 6]. To the best of our knowledge, only a few cases have been reported of a patient with unilateral complete ureteral duplication and ureteral stone [7]. We present a case of a hydronephrotic lower moiety of the left complete duplex system rather than the usual presentation of reflux hydronephrosis, caused by left midureteric stone in an adult female with a unilateral left duplex renal system which was asymptomatic for more than thirty years.

## 2. Case Report

### 2.1. Patient Information

A 35-year-old female patient presented with left flank pain in the last three years. The pain was a dull aching type that did not prevent her from accomplishing her daily work and not radiating without aggravating or relieving factors. The patient was visited by a primary health care personnel and diagnosed with renal stone without improvement with medical therapy. The patient had hypertension and was controlled with oral medication ten days ago.

### 2.2. Clinical Findings

On physical examination, her vital signs were normal. In the abdominal examination, she only had mild left costovertebral angle tenderness.

### 2.3. Diagnostic Assessment

**Figure 1:**
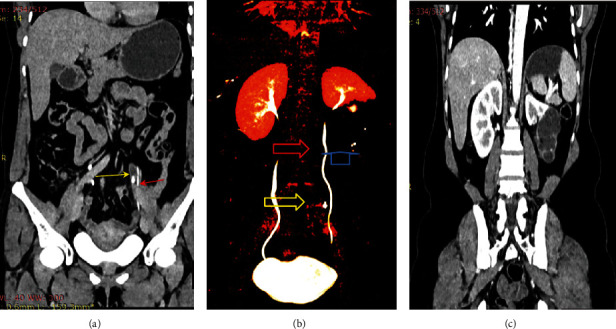
Abdominal CT scan showed the following: (a) coronal view shows both functioning (red arrow) and dilated ureter with proximal dilatation (yellow arrow); (b) 3D CT construction showing midureteric stone (yellow arrow), nonobstructed ureter with complete urinary drainage from upper segment (red arrow), and hydronephrotic segment (blue arrow); (c) contrast film showing lower pole nonfunctioning and hydronephrotic kidney.

Blood tests revealed a total white blood cell count of 9 × 10 [3]/ml, hemoglobin of 14.3 g/dl, blood urea nitrogen of 14 mg/dl, and creatinine of 0.52 mg/dl. Ultrasound of the kidney showed left multiple well-defined anechoic lesions with distal acoustic enhancement (fluid-filled structure) seen on the left kidney mid and lower pole. Computerized tomography (CT) scan of the urinary system (CT urography) showed normal excreting of the right kidney and ureter with no obstruction. The left kidney duplex pelvis with two complete ureters draining into the bladder where the lower kidney draining ureter was dilated to the level of the fourth lumbar vertebrae with obstructed stone measuring 7 mm in diameter (Figure 1).

**Figure 2:**
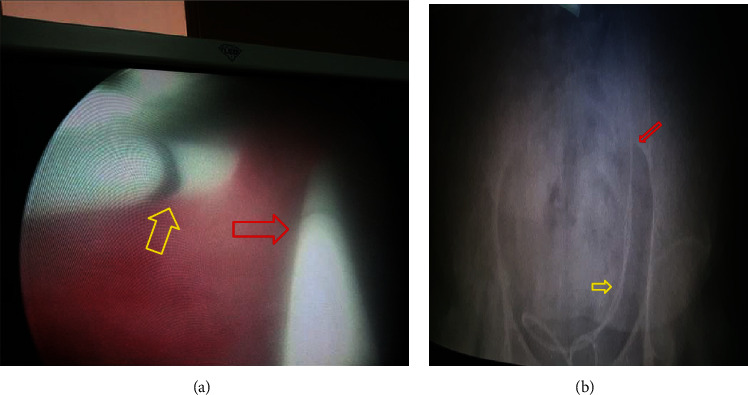
(a) Preoperative cystoscopy showing the two left ureteric orifices where the medial is allowing JJ stent placement (yellow arrow) and the lateral one is intubated with a ureteric catheter (red arrow) (obstructed ureter). (b) KUB shows the two catheters where the lateral could not go beyond the mid sacrum because of ureteric stone (yellow arrow) and normally placed (red arrow) JJ stent.

Diagnostic cystoscopy showed a single right ureteric orifice and two left ureteric orifices where the nonobstructed ureteral orifice appears medial where JJ stent Fr 6 size was placed without difficulty. The obstructed ureteral orifice appears lateral and caudal where the ureteric catheter advanced till to the obstructed part (midureter) due to impacted stone with no association with ureterocele (Figure 2). A diagnosis of a complete duplex kidney with lower moiety hydronephrosis was made, and the patient was prepared for laparotomy. Ideally, laparoscopic surgery was preferred, but we did open surgery due to the low facility.

### 2.4. Therapeutic Interventions

**Figure 3:**
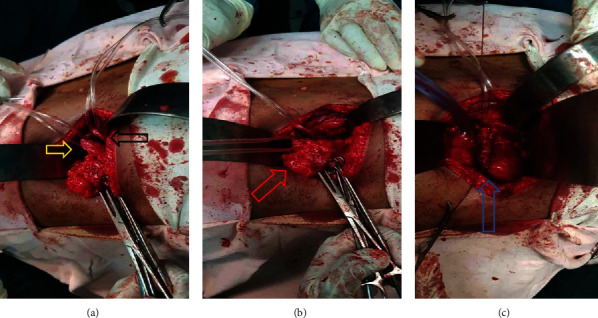
Intraoperative imaging. (a) Dilated proximal ureter draining lower moiety (yellow arrow), another ureter draining upper moiety (black arrow). (b) Hydronephrotic segment (red arrow). (c) Repair of the upper segment after lower pole resection (blue arrow).

Preoperative antibiotic (ceftriaxone: 1 g) was administered. Then, the abdomen was entered through a left subcostal flank incision under general anesthesia. Retroperitoneal space was entered, and the left kidney was identified after Gerota's fascia was opened. The lower pole of the kidney was hydronephrotic and looked like a bag of fluid. The two ureters were identified and found with the upper pole insertion, and the other was draining the lower pole (Figure 3(a)).

The lower segment vasculature and upper moiety blood supply were identified, and the left partial nephrectomy was done (Figures 3(b) and 3(c). The lower pole draining ureter was ligated after stone removal. An intra-abdominal drain was left in situ, and the abdominal wall was closed in layers.

### 2.5. Follow-Up and Outcome

The patient had a smooth postoperative course following the surgery. The drainage tube was removed during a 36-hour postoperative period. The patient was discharged on her fifth postoperative day. On the 15th postoperative day, the patient was found to have a good condition without complications.

## 3. Discussion

The presence of two pelvicalyceal systems defines the duplex kidney system; the condition is a known congenital anomaly of the urinary tract [6, 8].

Most of them are incidental findings on routine imaging for other compliance; if it is symptomatic, it is caused by obstruction, reflux, or calculi alone or in combination with other associated anomalies, including ureterocele or ectopic ureter [4]. Females are more likely to have unilateral or bilateral duplex renal systems [9].

During embryonic development, two ureteral buds rarely develop independently from a single mesonephric duct, resulting in a duplex kidney with ureteral duplication [7, 10].

Even rarer is unilateral complete ureteral duplication with single renal parenchyma drained by two pyelocaliceal systems [4]. Urinary stone formation is possible comorbidity in patients with a duplex system. There have been few reports of patients with duplex systems and urinary stones who also have ureterocele or collecting system obstruction [7, 11].

Complete duplication is usually unilateral. Nevertheless, bilateral cases are also reported [4]. One of the complications associated with duplication is obstruction, which could be calculus (anywhere along the ureter) or noncalculus obstruction at a confluence of the ureter or bladder insertion [4, 12]. Our patient presented with ureteral calculus obstruction of the lower moiety draining ureter at the level of the mid ureter.

According to Meyer-Weigert-Rule, the upper pole is generally seen as ectopic and therefore dysplastic due to obstruction, whereas the lower pole is related to vesicoureteral reflux [13]. In our case, the patient had a left complete duplicated kidney with a lower draining ureter and left lower hydronephrotic segment, and a left mid ureteric stone caused a dilatation in the proximal part of the left kidney.

Amis et al. reviewed 11 cases of obstruction involving the lower pole moiety of kidneys. There were complete duplications in seven cases. The pathologic entities caused by ureteropelvic junction obstructions in 2 cases, bladder tumor in 2 cases, ectopic upper pole ureterocele compressing and obstructing the ureteral orifice to the lower pole in one case, and ureteral stones in two cases (located at the mid and ureterovesical junction) [5]. Similarly, our patient had a midureteric stone which caused prolonged obstruction unnoticed for an extended period.

The first modality used was ultrasound showing our patient's multicystic mid- and lower pole segments. Later, other imaging modalities used to confirm or rule out conditions must be held. These include voiding cystourethrography, which is used to rule out reflux. Intravenous urography IVP and CT urography to confirm the duplex system and other obstruction causes, and nuclear imaging to realize the split function and level of obstruction [4, 14]. In our case, CT urography was performed and showed a nonfunctional lower pole of the left kidney and dilated ureter.

Another option for clarifying the anatomy of duplication anomalies is preoperative cystoscopy combined with a retrograde ureteropyelogram prior to surgical correction [15].

Management depends on the cause of obstruction and the cortical loss in the renal moiety [4]. The majority of cases can be managed endoscopically and laparoscopically and through open surgery in resource-limited facilities [16]. The role of treatment is to relieve the obstruction cause or relieve the pain by removing part of the kidney (partial nephrectomy) [4]. In our patient, partial nephrectomy was performed. Several published reports mentioned a similar procedure [9, 17, 18].

Our patient might have benefited from a simple endoscopic procedure if the diagnosis had been made sooner. However, her delayed presentation to appropriate care due to her primary healthcare providers' low suspicion index resulted in this unfortunate complete renal moiety parenchymal loss; a similar result was reported by Anyimba et al. [4]

## 4. Conclusion

The previously asymptomatic duplex renal system may develop symptoms later in life. When identified without symptoms or evidence of renal function compromise, the integrity of the renal moieties may have to be closely monitored for the rest of its life. A thorough evaluation with a high index of suspicion is required in individuals presenting with flank pains to quickly identify missed duplex renal systems and duplex system associated disorders quickly and thus prevent the attendant renal moiety parenchymal loss.

## Data Availability

All data materials related to the case report are included in the manuscript.

## References

[1] Sarma D., Pratihar S. K., Rajeev T. P., Barua S. K., Bagchi P. K., Phukan M. (2019). Duplex kidney anomalies and associated pathology: a single centre retrospective review. *International Surgery Journal*.

[2] Lynch J. O., Cox A., Rawal B., Shelmerdine S., Vasdev N., Patel A. (2016). Bilateral obstruction of bilaterally duplicated collecting systems requiring upper and lower moiety drainage. *The Annals of The Royal College of Surgeons of England*.

[3] Privett J. T., Jeans W. D., Roylance J. (1976). The incidence and importance of renal duplication. *Clinical radiology*.

[4] Anyimba S. K., Nnabugwu I. I., Nnabugwu C. A. (2021). Obstructed right upper moiety in a bilateral partial duplex renal system in an adult. *Annals of African Surgery*.

[5] Amis E. S., Cronan J. J., Pfister R. C. (1985). Lower moiety hydronephrosis in duplicated kidneys. *Urology*.

[6] Aeron R., Sokhal A. K., Kumar M., Sankhwar S. (2017). Giant hydronephrosis in a case of ureterocele with a duplex system: an entity yet not reported. *BML Case Reports*.

[7] Karakose A., Aydogdu O., Atesci Y. Z. (2013). Unilateral complete ureteral duplication with distal ureteral stone: a rare entity. *Canadian Urological Association Journal*.

[8] Başdaş C., Çelebi S., Özaydın S. (2016). Unusual presentation of duplex kidneys: ureteropelvic junction obstruction. *Advances in Urology*.

[9] Theophanous R. G., Limkakeng A. T., Broder J. S. (2020). Duplicated or ectopic renal collecting system in two adult emergency department patients. *The Journal of Emergency Medicine*.

[10] Prakash R. T., Venkatiah J., Bhardwaj A. K., Singh D. K., Singh G. (2011). Double ureter and duplex system: a cadaver and radiological study. *Urology Journal*.

[11] Ghobashy A., El-Shazly M., Lari A. (2012). A case of complete renal duplex with h-shaped ureter. *Case Reports in Urology*.

[12] Liu W., Zhang L., Ma R., Wu R. (2016). The morphology and treatment of coexisting ureteropelvic junction obstruction in lower moiety of duplex kidney. *International Journal of Surgery*.

[13] Darr C., Krafft U., Panic A., Tschirdewahn S., Hadaschik B. A., Rehme C. (2020). Renal duplication with ureter duplex not following Meyer-Weigert-rule with development of a megaureter of the lower ureteral segment due to distal stenosis - a case report. *Urology Case Reports*.

[14] Haliloglu M., Akpinar E., Akhan O. (2002). Lower-pole ureteropelvic junction obstruction with abnormal rotation in duplicated system. *European Journal of Radiology*.

[15] Bruno D., Delvecchio F. C., Preminger G. M. (2000). Successful management of lower-pole moiety ureteropelvic junction obstruction in a partially duplicated collecting system using minimally invasive retrograde endoscopic techniques. *Journal of Endourology*.

[16] HosseiniSharifi S. H., Nabavizadeh B., Mozafarpour S., Kajbafzadeh A. M. (2017). Laparoscopic selective clipping of upper moiety vasculature and ureter without partial nephrectomy: a novel technique for pediatric urinary incontinence due to ectopic ureter associated with poor functioning upper renal moiety. *Journal of Pediatric Urology*.

[17] Esposito C., Escolino M., Autorino G. (2021). Laparoscopic partial nephrectomy for duplex kidneys in infants and children: how we do it. *Journal of Laparoendoscopic & Advanced Surgical Techniques*.

[18] Chen C. T., Wang S. F. (2020). A case of left duplex kidney with hydronephrosis mimicking a left renal cyst in a 29-year-old woman. *The American Journal of Case Reports*.

